# Contributions of the Endothelium to Vascular Calcification

**DOI:** 10.3389/fcell.2021.620882

**Published:** 2021-05-17

**Authors:** Li Zhang, Jiayi Yao, Yucheng Yao, Kristina I. Boström

**Affiliations:** ^1^Division of Cardiology, David Geffen School of Medicine at UCLA, Los Angeles, CA, United States; ^2^UCLA Molecular Biology Institute, Los Angeles, CA, United States; ^3^UCLA Jonsson Comprehensive Cancer Center, Los Angeles, CA, United States

**Keywords:** vascular endothelium, endothelial cells, vascular calcification, endothelial-mesenchymal transition, bone morphogenetic protein

## Abstract

Vascular calcification (VC) increases morbidity and mortality and constitutes a significant obstacle during percutaneous interventions and surgeries. On a cellular and molecular level, VC is a highly regulated process that involves abnormal cell transitions and osteogenic differentiation, re-purposing of signaling pathways normally used in bone, and even formation of osteoclast-like cells. Endothelial cells have been shown to contribute to VC through a variety of means. This includes direct contributions of osteoprogenitor cells generated through endothelial-mesenchymal transitions in activated endothelium, with subsequent migration into the vessel wall. The endothelium also secretes pro-osteogenic growth factors, such as bone morphogenetic proteins, inflammatory mediators and cytokines in conditions like hyperlipidemia, diabetes, and renal failure. High phosphate levels caused by renal disease have deleterious effects on the endothelium, and induction of tissue non-specific alkaline phosphatase adds to the calcific process. Furthermore, endothelial activation promotes proteolytic destruction of the internal elastic lamina that serves, among other things, as a stabilizer of the endothelium. Appropriate bone mineralization is highly dependent on active angiogenesis, but it is unclear whether the same relationship exists in VC. Through its location facing the vascular lumen, the endothelium is the first to encounter circulating factor and bone marrow-derived cells that might contribute to osteoclast-like versus osteoblast-like cells in the vascular wall. In the same way, the endothelium may be the easiest target to reach with treatments aimed at limiting calcification. This review provides a brief summary of the contributions of the endothelium to VC as we currently know them.

## Vascular Calcification and the Endothelium

Vascular calcification (VC) is a frequent complication of cardiovascular disease (Sage et al., 2010; Marulanda et al., 2014) that increases morbidity and mortality and constitutes a significant obstacle in interventions and surgeries (Polonsky et al., 2010; Yutzey et al., 2014; Gepner et al., 2015). It occurs commonly as media sclerosis in vasculopathy caused by diabetes, chronic kidney disease (CKD), hypertension and aging, and as lesion calcification in atherosclerotic plaques (Luscher et al., 2003; Giachelli, 2009; Shanahan et al., 2011; Boström, 2016). Clinical studies have reported a high prevalence of arterial calcification that increases with age and is seen in more than 90 and 67% of men and women over 70 years of age, respectively, (Liu et al., 2015). The physiological changes resulting from the arterial stiffening in media sclerosis contributes to systolic hypertension and congestive heart failure, whereas the lesion calcification may directly contribute to plaque instability and increase the complexity of interventions in coronary obstructions.

**FIGURE 1:**
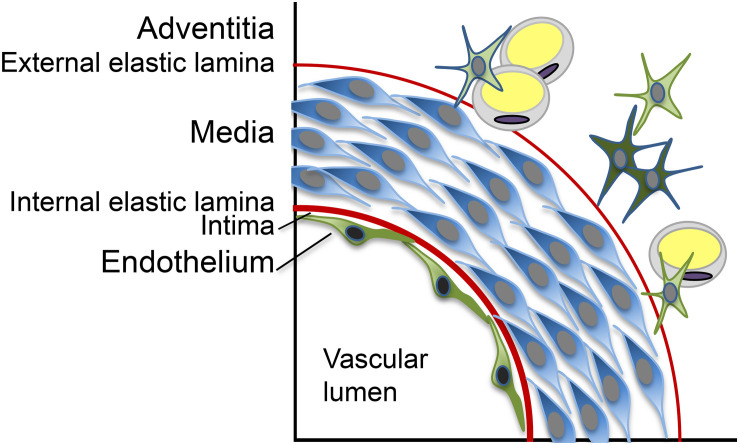
Schematic representation of the layers in the vascular wall.

Vascular calcification is widely accepted to be an active and regulated process, which shares many similarities with bone formation and involves abnormal cells transitions, osteogenic differentiation, and signaling pathways frequently used in bone (Sage et al., 2010; Boström, 2016). The vascular media is the most common location of calcific lesions, but calcification can appear as a calcified internal elastic lamina (IEL), exophytic calcification extending into the vascular lumen or generalized calcification affecting several layers of the vascular wall (Boström, 2016; Figure 1; schematic layers). Although most attention has been given to the vascular media, all vascular layers are likely to contribute to the VC in different ways. The role of the vascular endothelium, in particular, has come under increased scrutiny during the past decade. The endothelium is the innermost layer of the vascular tube and serves as an interface between the blood stream and the rest of the vascular wall. Normally, this exceedingly thin structure consists of a single layer of endothelial cells (ECs) and is tasked with various responsibilities such as the maintenance of non-thrombogenic surfaces and quiescence in the vascular wall. The effort is aided by the presence of the IEL on which the endothelium rests. The endothelium is also tasked with responding to different stimuli that are generated in the local environment or delivered by the circulation. As a result, the endothelium can exist in various states of activation, including inflammatory, angiogenic and osteogenic phenotypes, and thereby act as a mediator of vascular disease.

The characteristics of the inflammatory and angiogenic endothelial phenotypes have been extensively studied in atherosclerosis and angiogenesis and are reviewed in Gimbrone and Garcia-Cardena (2016) and Eelen et al. (2020). However, ECs with osteogenic phenotype and their role in calcific vasculopathy are less well understood. Here, we provide a brief summary on what we know so far about the ways the endothelium contributes to the calcific process.

## Endothelial-Mesenchymal Transitions

**FIGURE 2:**
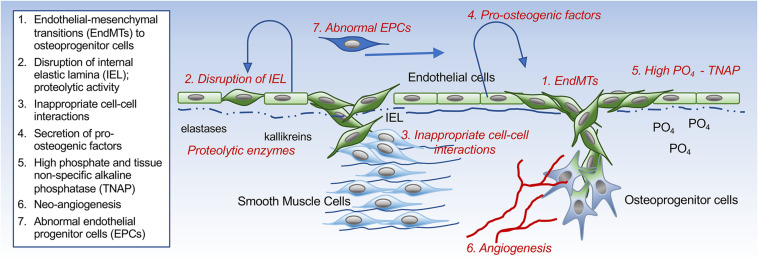
Schematic drawing. Different ways the endothelium can influence the development of vascular calcification.

The endothelium has the ability to contribute osteogenic progenitor cells to VC through cellular transitions (Figure 2; schematic figure). Endothelial-mesenchymal transitions (EndMTs) represent a cellular reprogramming where ECs acquire mesenchymal cell characteristics while the endothelial characteristics diminish. Even though a molecular definition of EndMTs is not yet fully established, several reviews are helpful in outlining the basis and criteria for EndMTs (Li et al., 2018; Kovacic et al., 2019). These include an enhanced cellular capacity for invasion, migration and contraction, and the expression of markers linked to the triggering of EndMTs such as Snail, Slug, Twist, Krüppel-like factor 4, and N-Cadherin (Yao et al., 2015; Li et al., 2018; Kovacic et al., 2019; Ma J. et al., 2020). Markers that are commonly used to monitor the endothelial lineage and the emergence of fibroblastic and osteogenic mesenchymal characteristics in ECs are listed in Table 1 (Medici et al., 2010; Yao et al., 2015; Evrard et al., 2016; Li et al., 2018). It is not fully understood whether the cells proceed through a defined stem cell-like stage prior to diverting into fibroblastic or osteogenic lineage. It is possible that the ECs only undergo partial EndMTs without reaching terminal mesenchymal differentiation as has been observed in for example glomerulosclerosis (Kato et al., 2014; Li et al., 2018). Both endothelial, mesenchymal and osteogenic markers would then be co-expressed by the intermediate cells, and may be enough to influence VC. Partial EndMTs may be easier to reverse to the original endothelial lineage.

**TABLE 1:** Lineage markers commonly used in studies of endothelial-mesenchymal transitions and vascular calcification.

Endothelial lineage
CD31 (also known as PECAM1) vWF (von Willebrand factor) Cadherin 5 (also known as VE-cadherin) VEGFR2 (vascular endothelial growth factor receptor 2; also known as KDR or Flk-1)

**Fibroblastic mesenchymal lineage**

COL1A1 (α1 type I collagen) COL1A2 (α2 type I collagen) SOX9 (SRY-box 9) PDGFRα (platelet-derived growth factor-α) TCF21 (transcription factor 21) Vimentin FSP1 (fibroblast-specific protein 1; also known as S100-A4) DDR2 (discoidin domain-containing receptor 2) THY1 (Thy-1 membrane glycoprotein; also known as CD90)

**Myofibroblast lineage**

αSMA (α smooth muscle actin) Periostin

**Osteogenic lineage**

Rux2 (Runt-related transcription factor 2; also known as Cbfa1) Osterix (also known as Sp7) COL1A1 (α1 type I collagen) Alkaline phosphatase expression and activity Osteocalcin Osteopontin Fibronectin Calcium mineral

## EndMTs Discovered as a Source of Calcifying Cells in Fibrodysplasia Ossificans Progressiva

The concept that the endothelium in the systemic arteries contributes osteoprogenitor cells to VC came as a logical extension from the studies on fibrodysplasia ossificans progressiva (FOP). In 2010, Medici et al. (2010) reported that microvascular ECs with activating mutations in activin receptor-like kinase 2 (ALK2) were converted into multipotent stem cell-like cells as shown by TEK receptor tyrosine kinase (*Tie2*)−*Cre* lineage tracing (Medici et al., 2010). ALK2 is a BMP type 1 receptor encoded by the *Acvr1* gene that is essential for the development of multiple tissues and organs, such as bones, muscles and brain. A number of BMPs and activins bind to ALK2 (Sekimata et al., 2020), among them BMP4 and 6, which are commonly expressed in the endothelium and participate in angiogenesis and cell proliferation (Bautch, 2019). In patients with FOP caused by mutated ALK2 receptors and mice with corresponding mutations, the converted ECs were shown to participate in the calcific soft tissue lesions that characterize this disorder (Medici et al., 2010; Sekimata et al., 2020). Furthermore, their studies showed that BMP4 and transforming growth factor (TGF)β2, both members of the TGFβ superfamily of growth factors, mimicked the effect of the activating ALK2 mutations *in vitro* (Medici et al., 2010). Indeed, EndMTs required activation of both ALK2 and ALK5 by BMP4 and TGFβ2, respectively, with subsequent signaling through SMAD5 (BMP-activated), and SMAD2 (TGFβ-activated; Medici et al., 2010). Interestingly, binding of BMP7 to ALK2 did not recruit ALK5, and therefore activated only SMAD5 without triggering EndMTs (Medici et al., 2010). Moreover, Lin H. et al. (2019) showed that Activin A, another member of the same growth factor family, also activated abnormal BMP signaling through the mutated ALK2. This occurred despite Activin A normally signaling through SMAD2/3 and inhibiting BMP signaling. Together, the studies provided evidence that ECs might contribute osteoprogenitor cells to the ectopic tissue calcification through EndMTs, and that abnormal signaling among the TGFβ growth factors mediate such transitions.

## EndMTs in Vascular Calcification

Evidence from several mouse models suggest the presence of endothelial dysfunction and EndMTs in VC (Kajimoto et al., 2015; Malhotra et al., 2015; Yao et al., 2015; Evrard et al., 2016; Sanchez-Duffhues et al., 2018). The matrix Gla protein null (*Mgp*^–/–^) mouse, a VC model where loss of MGP results in rapid and progressive calcification and an abnormal endothelium (Luo et al., 1997; Yao et al., 2015). MGP is a small matrix protein with calcium-binding capacity that binds several BMPs involved in endothelial and osteogenic biology such as BMP2, 4, and 7 (Yao et al., 2008). MGP is highly expressed in the mature endothelium and essential for its integrity in the systemic arteries that are prone to developing VC. Studies have shown that in absence of MGP, the IEL is progressively destroyed through excessive proteolysis, causing the ECs to lose their anchoring and become susceptible to EndMTs (Yao et al., 2015). As part of this process, the ECs co-expressed endothelial and mesenchymal or osteogenic markers in the calcific lesions (Yao et al., 2013, 2015; Malhotra et al., 2015), as determined by lineage tracing using the *Tie2*-Green fluorescent protein (*Gfp*) transgenic mouse and co-immunofluorescence (Yao et al., 2013). The response to loss of MGP was mimicked in human aortic ECs stimulated by BMP4 or high glucose *in vitro*, and was limited by the BMP inhibitor Noggin (Yao et al., 2013). In these studies, the transcription factor SRY (sex determining region Y)-box 2 (Sox2) was identified as an active regulator of EndMTs that led to osteogenic ECs, acting downstream of BMP (Yao et al., 2015; Sanchez-Duffhues et al., 2018). Sox2 was already known as a potent driver of fate conversion and direct reprogramming in somatic cells as one of the four pluripotency genes (Julian et al., 2017). Endothelial deletion of the *Sox2* gene limited both EndMTs and VC in *Mgp*^–/–^ mice (Yao et al., 2015). The involvement of Sox2 in the regulation of EndMTs represent a novel aspect of its function.

Additional evidence supporting the concept of BMP activation and EndMTs in vascular disease was derived from atherosclerotic *Apoe*^–/–^ mice and diabetic *Ins2*^Akita/+^ mice [(Sanchez-Duffhues et al., 2018) review]. Both *Apoe*^–/–^ and *Ins2*^Akita/+^ mice have enhanced endothelial BMP4 expression in response to hyperglycemia and hyperlipidemia, respectively, which mimics the loss of BMP inhibition and allows an emergence of EndMTs and VC. When diabetic *Ins2*^Akita/+^ mice are crossed with *Mgp* transgenic mice, BMP4 expression is suppressed (Bostrom et al., 2010), suggesting that the augmented MGP level was sufficient to limit the BMP activity. The studies suggest that the BMP4-MGP balance could serve as a point of influence for factors that affect VC, such as warfarin that interferes with the necessary gamma-carboxylation of the MGP protein (Yao et al., 2008). As the transitioned ECs migrate into the vascular wall, they may be exposed to BMP2, which is highly induced in calcified vascular lesions (Sweatt et al., 2003) and would promote calcification of osteogenic ECs.

It should be noted that at least two of the studies on EndMTs in tissue calcification used the *Tie2*-promoter for endothelial lineage tracing (Medici et al., 2010; Yao et al., 2013). More recent studies generally prefer the vascular endothelial (VE)-Cadherin (*Cdh5*)-promoter for endothelial lineage tracing and excision, even if expression of both Tie2 and VE-cadherin has been detected in small subpopulations of hematopoietic cells and could influence the lineage tracing (Kisanuki et al., 2001; Alva et al., 2006; Yao et al., 2013). A similar issue exists for the vascular smooth muscle cells (SMCs). A transition of SMCs to osteogenic cells was also reported in the aorta of the *Mgp*^–/–^ mice, as determined by lineage tracing using the *Sm22α-Cre* promoter (Speer et al., 2009). However, the SM22*α* protein is not unique to the SMCs and is expressed in a number of mesenchymal cells such as myofibroblasts, pericytes, and even ECs undergoing EndMTs (Ding et al., 2004; Kokudo et al., 2008; Wirz et al., 2008). It is therefore difficult to accurately assess what portion of the osteogenic cells in calcific lesions are derived from ECs versus medial SMCs. Combinations of lineage tracers or results from single cell sequencing may be able to provide a better understanding of this issue in the future.

In a set of *in vitro* studies, Yung et al. (2015) showed that BMP6 and oxidized low density lipoproteins (oxLDL) triggered osteogenic differentiation in bovine aortic ECs, both independently or synergistically. The process was abrogated by scavenging of the reactive oxygen species (ROS) generated in response to oxLDL or by inhibiting the BMP receptors (Yung et al., 2015). In addition to ROS, inflammatory mediators such as the tumor necrosis factor (TNF)−α and interleukin (IL)−1β were reported to induce EndMTs in human aortic ECs and sensitize them to BMP9, a potent osteoinductive BMP in the circulation (Sanchez-Duffhues et al., 2019). Interestingly, lack of primary cilia was shown to further sensitize the endothelium to undergo BMP-dependent osteogenic differentiation, as mediated by β-catenin-induced transcription factor SLUG (Sanchez-Duffhues et al., 2015). Thus, synergy between oxidative stress, inflammation and BMP activity enhances the involvement of the endothelium in the calcific process.

Although TGFβ and BMP signaling have been shown to act in conjunction to trigger EndMTs (Medici et al., 2010), the connections between them in the endothelium is an understudied area. There are several ways they could interact in the transition from the early stages of EndMTs to the emergence of osteogenic phenotypes. One possibility is that the ECs take on the characteristics of myofibroblasts, which are activated by TGFβ (Zent and Guo, 2018), and then undergo BMP-mediated calcification (Yang et al., 2020). Other possibilities could involve alterations in the transcriptional regulation of endothelial SMADs or complex formation of the TGFβ/BMP receptors.

## Aortic Induction of ALK1 Might Facilitate BMP9-Mediated Osteoinduction

Activin receptor-like kinase (ALK)1 is best known as an endothelial receptor that promotes maturation and quiescence in the normal endothelium (David et al., 2008). ALK1 has also been discovered to participate in the uptake of LDL in ECs (Kraehling et al., 2016). BMP9 signaling is mediated by ALK1 and potentially ALK2 with effects on the endothelial lineage as well as osteoinduction (David et al., 2008; Lamplot et al., 2013). Interestingly, Sanchez-Duffhues et al. (2019) showed that the osteoinductive effect could be mitigated by suppressing the BMP type 2 receptor (BMPR2) and JNK signaling (Sanchez-Duffhues et al., 2019), and Theilmann et al. (2020) showed that loss of BMPR2 drove a proliferative response to BMP9 in EC, which is a reversal of the usually response promoting cell maturation. Thus, the outcome of BMP9 signaling is likely to vary depending on the on the state of BMPR2 and downstream signaling.

**FIGURE 3:**
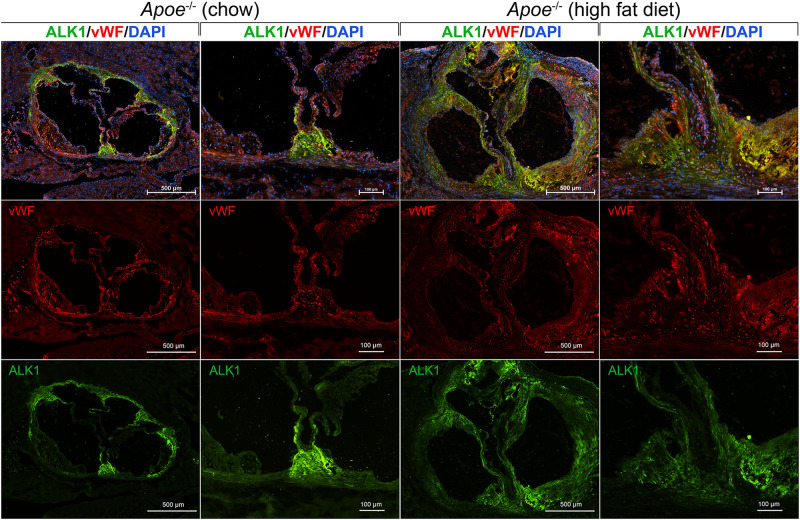
ALK1, a receptor for BMP9, is induced in the aorta and aortic valves of Apoe^−/−^ mice fed a high fat diet. *Apoe*^−/−^ mice were fed regular chow or high fat diet for 6 weeks. Histological sections were prepared from the proximal aorta that included the aortic valves. The sections were stained for activin receptor-like kinase 1 (ALK1; green fluorescence) and von Willebrand factor (vWF; red fluorescence). DAPI was used for visualization of nuclei. The results showed a widespread induction of ALK1 in the thickened valves and aortic wall in the fat-fed *Apoe*^− /−^ mice, which would allow for enhanced BMP9 osteoinduction.

We examined the expression of ALK1 in the aorta and valves in 8-week old *Apoe*^–/–^ mice after fat-feeding. The *Apoe*^–/–^ mouse is a well-known model of atherosclerosis and aortic valve disease, and were fed standard chow (Diet 8604, Harlan Teklad Laboratory) or a high-fat/high-cholesterol diet (Western diet; Research Diets, diet #D12108) for 6 weeks. The aortas were prepared and stained for ALK1 and the endothelial marker von Willebrand factor (vWF) by immunofluorescence using the methodology and antibodies as described (Yao et al., 2010). 4′,6-Diamidino-2-Phenylindole (DAPI) was used for visualization of the nuclei. The results showed that ALK1 was highly induced in the aortic wall and the valves of the fat-fed mice (Figure 3). In chow-fed mice, ALK1 was expressed mostly in the aortic commissures, whereas vWF was detected along the valvular endothelium and the base of emerging atherosclerotic lesions. After fat-feeding, both ALK1 and vWF were widely expressed in the thickened valves and atherosclerotic lesions, but with limited co-expression, suggesting that ALK1 was expressed in both ECs and other valve cells and might allow for enhanced osteoinduction by circulating BMP9.

## Suppression of EndMTs and Calcification

Signaling pathways that counteract EndMTs and possibly calcification have been identified. The strong angiogenic activators vascular endothelial growth factor (VEGF) and basic fibroblast growth factor (bFGF, also referred to as FGF2) have been reported to counteract the EndMT process (Wang et al., 2016). Interestingly, bFGF is an activator of MGP expression (Stheneur et al., 2003), which might enhance its ability to suppress EndMTs. SPARC Related Modular Calcium Binding 2, which is also able to serve as an angiogenic factor (Rocnik et al., 2006), has been shown to limit mineralization in human umbilical vein ECs (HUVECs) during extended cell culture (Peeters et al., 2018). Furthermore, high-density lipoproteins, which is considered a beneficial factor in vascular disease, reduces EndMTs in human aortic ECs by interfering with TGF-β1 signaling (Spillmann et al., 2015). BMP7 limits EndMTs by avoiding activation of TGFβ signaling (Medici et al., 2010), and is able to prevent VC in chronic uremia in rats but not reverse already established calcification (Gravesen et al., 2018). However, loss of function mutations in the *Bmpr2* gene, which are linked to pulmonary arterial hypertension, cause an enhancement of TGFβ signaling and EndMT marker expression (Kovacic et al., 2019).

A number of drugs that are already in clinical use could potentially limit EndMTs and VC by interfering with TGFβ signaling [reviewed in Man et al. (2019)]. These include losartan, an angiotensin II receptor blocker widely used for systemic hypertension and aortic aneurysms, and macitentan, which is used for patients with pulmonary arterial hypertension. However, none has been tested for effects on VC.

## Disruption of the Internal Elastic Lamina Is Part of Vascular Pathology

The IEL is a layer of elastic tissue that forms a barrier between the endothelium and the vascular media (Figure 2; schematic figure). It might be seen as a stabilizing structure for both the vascular endothelium and for the innermost layer of the medial SMCs. Abnormalities of the IEL have been reported in several vascular disorders. For example, disruption, reduplication and calcification of the arterial IEL can be found in Buerger’s disease (also referred to as thromboangiitis obliterans), which has a strong link to tobacco use, and Kawasaki’s disease, a vasculopathy of unknown cause (Senzaki, 2006; Micheletti et al., 2008). In addition, abnormalities have been reported in VC due to the deficiency of ecto-5′-nucleotidase (NT5E, also referred to as CD73; Markello et al., 2011). CD73 is an extracellular enzyme that mediates the conversion of AMP into adenosine and inorganic phosphate (St Hilaire et al., 2011). Rupture of the aortic IEL has also been reported in rats during growth and aging (Osborne-Pellegrin et al., 2010). Interestingly, calcification exclusively involving the IEL has been reported in patients with HIV-associated vasculopathy and Buerger’s disease (Micheletti et al., 2008), but it is unclear how this highly localized process is triggered or if the endothelium is involved.

Activation of proteolysis has been reported in association with the development of VC. EndMTs in the *Mgp*^–/–^ mouse was associated with BMP4-mediated induction of elastase 1 and 2, and kallikrein 1, 5, and 6 (Yao et al., 2015), which ultimately caused the destruction of the entire aortic IEL (Yao et al., 2013, 2015). Protease inhibition by SERPINA1 or diisopropylfluorophosphate prevented the destruction. In the same mouse model, Beazley et al. (2013) reported induction of the major elastase adipsin and extensive fragmentation of elastic lamellae that preceded calcification. Elastin degradation products have been shown to enhance osteogenesis in myofibroblasts (Simionescu et al., 2007) and could affect both ECs and SMCs in this mouse model.

The progressive IEL destruction may disrupt normal interactions between the endothelium and the media allowing pro-calcific stimuli from the ECs to influence the medial cells (in addition to the various breakdown products). Indeed, it has been reported that exosomes released from glucose-treated HUVECs contain Notch3, an important receptor in cell differentiation, and are able to promote calcification in SMCs through the mammalian target of rapamycin signaling pathway (Lin X. et al., 2019). Similarly, other investigators have found that ECs exhibit osteogenic properties that depend on Notch signaling in co-cultures with aortic SMCs (Kostina et al., 2019). ECs were also able to promote calcification of aortic SMCs derived from spontaneously hypertensive rats, which was facilitated by endothelial matrix metalloproteinase (MMP)−2 and MMP−9 (Meng et al., 2018). This supports that the integrity of the IEL is a key factor in separating and protecting different vascular layers from disease.

## Pro-Calcific Factors Derived From the Endothelium

The endothelium is the first line of defense against circulating or local stimuli that could turn the endothelium pro-calcific. It can act as a source of growth factors of cytokines used for autocrine or paracrine signaling (Figure 2; schematic figure). A prominent example is endothelial BMP-induction, which is triggered by a variety of stimuli. OxLDL, mechanical stress, and estrogen all have an inductive effect on BMP2 (Cola et al., 2004; Osako et al., 2010; Su et al., 2011; Rutkovskiy et al., 2019), whereas shear stress, hyperglycemia, hyperlipidemia, and oscillatory shear stress enhance BMP4 (Sorescu et al., 2004; Csiszar et al., 2009). The cellular effects of BMP2 and BMP4 often overlap, at least *in vitro*. BMP4 is a strong activator of angiogenesis, proliferation and EndMTs (Medici et al., 2010; Bautch, 2019). However, BMP2 has been reported in endothelial microparticles after endothelial injury (Buendia et al., 2015), which could hasten the shift toward calcification in neighboring cells (Blaser and Aikawa, 2018). Indeed, micro-vesicles from the plasma of elderly subjects or senescent ECs have been found to have enhanced levels of BMPs at sites of calcification (Alique et al., 2017). It is interesting to speculate how changing the type of BMP delivery might enhance both the activity and the reach in the vascular wall. Additional findings supporting the importance of BMP were obtained when the small molecule LDN-193189 or the recombinant BMPR-IA/ALK3-FC chimera protein were used for inhibition of the BMP receptors. This resulted in a successful reduction of endothelial dysfunction and vascular osteogenesis in mice with CKD and *Mgp* gene deletion (Kajimoto et al., 2015; Malhotra et al., 2015).

## Pro-Calcific Effect of High Phosphate in the Endothelium

High serum phosphate is well known to promote VC in CKD and has been linked to endothelial dysfunction [reviewed in Scialla and Wolf (2014)] (Figure 2; schematic figure). CKD and uremia induce the expression of tissue-specific alkaline phosphatase (TNAP), which degrades extracellular pyrophosphate to phosphate ions, thereby causing pyrophosphate to lose its inhibitory effect on VC (Lomashvili et al., 2008). Hortells et al. (2017) found that expression of TNAP preceded the initial calcium deposition in the aortic wall, and was later followed by expression of other osteogenic factors such as Runx2 and BMP2. Systemic hyperphosphatemia has several adverse effects on the endothelium, such as apoptosis, stimulation of ROS production, impairment of the acetylcholine-stimulated release of nitric oxide, and limitation of the angiogenic phenotype (Scialla and Wolf, 2014). Furthermore, ECs exposed to high phosphate have been observed to promote VC in SMCs, at least in part mediated by phosphate-induced expression of IL-8 in the ECs. This in turn limited induction of osteopontin, a calcification inhibitor, in the SMCs (Bouabdallah et al., 2019). The hyperphosphatemic state in ECs has also been shown to induce global changes in protein phosphorylation and enhance the release of prothrombotic microparticles (Abbasian et al., 2015).

Savinov et al. (2015) showed that EC-specific overexpression of TNAP, as directed by the *Tie2*-promoter, enhanced the osteogenic potential of ECs and promoted VC (Savinov et al., 2015). Overexpression of TNAP in the “wicked high cholesterol” (WHC) mouse model resulted in an unusual course of coronary atherosclerosis, where the calcification preceded the lipid deposition (Romanelli et al., 2017). The calcific lesions nucleated in the subendothelial space of medium-sized arteries and induced neointimal growth. Interestingly, even though TNAP was overexpressed in almost every EC, the calcification foci were relatively sparse, and seemed to require the high fat diet to create the early calcification pattern (Savinov et al., 2015). It is possible that the atherogenic diet activates endothelial expression of pro-osteogenic factors, such as BMPs, ROS and inflammatory mediators. It is not clear whether TNAP induction occurs exclusively in the endothelium in CKD or other disease states, without also occurring in the SMCs. It is interesting that high alkaline phosphatase activity was discovered long ago in the endothelium in small arteries and arterioles during branching (Romanul and Bannister, 1962), making it clear that the endothelial role of TNAP is not yet fully understood (Goettsch et al., 2020).

## Potential Role of Neo-Angiogenesis in VC

The endothelium is essential in responding to tissues requiring angiogenesis for oxygen and nutrients during development and regeneration. This is also true for bone. Angiogenesis is an absolute requirement for bone formation (Peng et al., 2020), where the preceding cartilage has an important role in recruiting ECs by secreting VEGF. A specialized bone endothelium is a critical part in bone growth, bone remodeling and during bone homeostasis (Kusumbe et al., 2014; Ramasamy et al., 2014; Peng et al., 2020). These vessels, referred to as type H vessels, express high levels of Endomucin and CD31 and are instrumental in the coupling of angiogenesis and osteogenesis. The type H vessels depend on Notch and VEGF signaling (Kusumbe et al., 2014; Ramasamy et al., 2014) but are also regulated by factors such as platelet-derived growth factor (PDGF)-BB and hypoxia-inducible factor 1α (HIF-1α).

Neo-angiogenesis extends into the diseased vascular wall and is driven by angiogenic factors like VEGF. It promotes the progression of atherosclerosis and may cause intraplaque hemorrhages, plaque rupture and tissue ischemia (Hiyama et al., 2010). Capillary ingrowth has been observed in proximity to plaque calcification (Jeziorska et al., 1998), and could be a determinant of the mineralization in the vascular wall or serve as a conduit for osteoprogenitor cells (Collett and Canfield, 2005). Although neo-angiogenesis has been identified as a potential target for strategies aimed at atherosclerosis (Sedding et al., 2018), anti-angiogenic therapies have so far not been shown to modulate VC.

## Endothelial Progenitor Cells in VC

Endothelial cells or endothelial progenitor cells (EPCs) may be recruited from the circulation to locations conducive to calcification (Figure 2; schematic figure). Gossl et al. (2008) showed that a high percentage of EPCs expressed osteocalcin (OCN+) in patients with coronary atherosclerosis and might have a role in VC. A subsequent study demonstrated that patients with early coronary disease retained these OCN + EPCs within the coronary circulation, potentially enhancing the development of calcification (Gossl et al., 2010). Specific disease states are likely to influence the EPC. For example, patients with diabetes had a pro-calcific phenotype, significantly driven by inflammatory stimuli (Fadini et al., 2012), whereas patients with CKD and VC had a rise in endothelial microparticles and a reduction in the number of EPCs, suggesting that the endothelial repair process was impaired (Soriano et al., 2014). Other investigators found that the number of OCN + EPCs was positively related to the frequency of spotty calcification in patients with acute coronary syndrome (Zhang et al., 2015), suggesting that the role of EPCs in VC may ultimately depend on the patient’s co-morbidities.

## Role of the Valve Endothelium in Calcific Aortic Valve Calcification

Calcific aortic valve disease (CAVD) is the most prevalent form of aortic stenosis, and is characterized by progressive fibro-calcific remodeling of the aortic valve leaflets (Lindman et al., 2016; Goody et al., 2020).

Population-based studies in developed countries have reported a high prevalence of CAVD that markedly increases over 65 years of age, and is around 3.4% for individuals over 75 years of age (Lindman et al., 2016), three quarters of which present with symptoms such as angina, congestive heart failure and syncope. The treatment for CAVD includes surgical and transcatheter aortic valve replacement, with no efficient medical treatment currently available. Similar to VC, the progressive calcification and fibrous thickening of aortic valves are actively regulated processes with similarities to chondrogenic and osteogenic differentiation.

During embryogenesis, EndMTs and crosstalk between ECs and the developing myocardium are instrumental for cardiac valve morphogenesis. There is also evidence suggesting that cellular reprogramming of the valvular ECs (VECs) occurs in CAVD (Ma X. et al., 2020). Results from Paranya et al. (2001) suggested that ECs derived from adult aortic valves could transition to a mesenchymal phenotype when stimulated by TGF-β or low levels of serum without addition of bFGF. This was supported *in vivo* by the presence of a cell population in human aortic valves that expressed both the endothelial marker CD31 and the SMC marker α-smooth muscle actin (α-SMA; Paranya et al., 2001). Later the same group showed that the mitral valves contained ECs with multilineage mesenchymal differentiation potential, including osteogenic differentiation (Wylie-Sears et al., 2011). Mahler et al. (2013) also reported a cell population of subendothelial cells co-expressing CD31 and α-SMA in calcific human aortic valves that further supports the existence of valvular EndMTs.

The valvular endothelium covers both the aortic (fibrosa) and the ventricular (ventricularis) side of the aortic leaflet, where the aortic side is most exposed to pro-osteogenic factors due to differences in shear stress. Simmons et al. (2005) reported a spatial heterogeneity of the valvular endothelial phenotype with distinct phenotypes on the two sides of the valve, which could explain the propensity for calcification on the aortic side. Later studies further showed that the valvular endothelium had distinct transcriptional differences, and should therefore be treated as a separate endothelial entity (Butcher et al., 2006). The porcine valvular endothelium had a more chondrogenic-oriented transcription profile when compared to the aortic endothelium (Butcher et al., 2006). Cadherin 11, which is linked to epithelial-mesenchymal transitions (Chen et al., 2021), was expressed in the VECs but not in the aortic ECs. However, BMP4 was expressed in both the aortic endothelium and the fibrosa side of the valve (Butcher et al., 2006), suggesting that BMP4 was in locations where it could trigger EndMTs. EndMTs occurring in the valves are also influenced by matrix stiffness and Wnt/β-catenin signaling (Zhong et al., 2018). Thus, even though conceptually similar, VC versus CAVD are likely to develop along distinct pathways.

Valvular ECs can serve as precursors of valvular interstitial cells (VICs; Bischoff and Aikawa, 2011), which in turn can take on an osteoblastic phenotype and enhance valve calcification. It has also been suggested that the VICs play a role in limiting EndMTs and osteogenesis in the VECs (Hjortnaes et al., 2015), which would support the importance of reciprocal VEC-VIC interactions for valve maintenance. Another example of VEC-VIC interactions include the VIC-mediated stimulation of VECs to invade the valves and form angiogenic networks through activation of Angiopoietin1-Tie2 signaling (Arevalos et al., 2016). In turn, the VECs influence the VICs by serving as a source of oxidative stress in response to TNF-β (Farrar et al., 2015). The response of the ECs, however, may not always be predictable as the valves age. A reduced regenerative capacity and senescence of VECs, in combination with low levels of EPCs, have been proposed to underlie the destruction of the valvular endothelium (Matsumoto et al., 2009). Overall, further studies of cellular transitions in the early and late stages of CAVD will be required to fully understand the unique features of the valve calcification.

## Loss of MGP in the Aortic Valves Induces BMP Activity and SOX2 Expression

As earlier stated, MGP has a powerful anti-calcific effect in the elastic arteries and is strongly expressed in the vascular endothelium (Luo et al., 1997; Yao et al., 2013). Interestingly, Hjortnaes et al. (2015) found in human aortic VECs that shear stress-activated Notch1 signaling up-regulated the expression of endothelial MGP, but down-regulated osteoblast-like gene networks (White et al., 2015). Theodoris et al. (2015) similarly found that Notch signaling was involved in the regulation of the *Mgp* gene using a system biology model that made use of a *Notch1* mutation, human-induced pluripotent stem cells and fluid flow.

**FIGURE 4:**
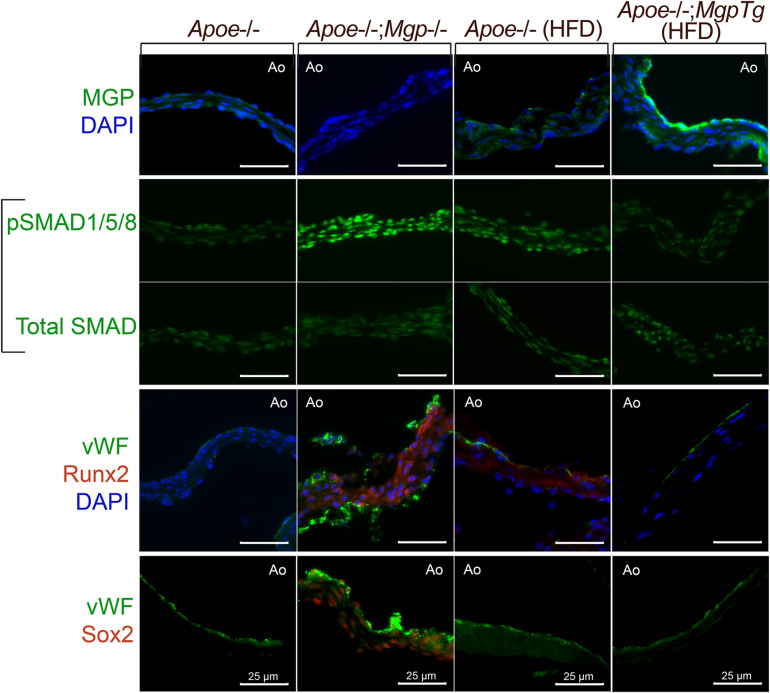
Aortic valves from Apoe^− /−^ mice with loss− or gain-of-function of Mgp. Aortic valves from *Apoe*^− /−^ and *Apoe*^− /−^; *Mgp*^− /−^ mice, and from *Apoe*^− /−^ mice and *Apoe*^− /−^; *Mgp*^*tg/wt*^ mice fed a HFD (16 weeks) were stained for MGP, phosphorylated (p)SMAD1/5/8, total SMAD, von Willebrand factor (vWF), Runx2, or Sox2 as indicated. When present, MGP was detected on the aortic side of the valve leaflet. Absence of MGP activated pSMAD1/5/8 and expression of Runx2 and Sox2, whereas excess MGP diminished pSMAD1/5/8, Runx2, and Sox2. Ao; aortic side (always facing up), Tg; transgene All bars are 25 μm.

We examined whether loss- or gain-of-function of MGP would affect SMAD signaling and osteogenic markers in the aortic valves in mice. We prepared histological sections from the aortic valves from *Apoe*^–/–^ or *Apoe*^–/–^;*Mgp*^–/–^ mice (with *Mgp* gene deletion; Yao et al., 2010) that had been fed standard chow for 16 weeks, and *Apoe*^–/–^ and *Apoe*^–/–^;*Mgp*^*tg*/*wt*^ mice (with a *Mgp* transgene; Yao et al., 2010) that had been fed a Western for 16 weeks. As reported, the *Apoe*^–/–^;*Mgp*^–/–^ mice are unable to tolerate a high fat diet (Yao et al., 2010). The sections were stained with immunofluorescence as described using the same antibodies for MGP, pSMAD1/5/8, total SMAD, Runx2 (Cbfa1), and Sox2 as in Yao et al. (2010, 2013). The results showed that the MGP protein was detected mainly on the aortic side of the valves (Figure 4, top row), similar to BMP4 in Butcher et al. (2006). Activated phosphorylated (p)SMAD1/5/8 was detected in the *Apoe*^–/–^;*Mgp*^–/–^ mice, consistent with enhanced BMP activity (Figure 4, middle rows). Furthermore, enhanced staining for the osteogenic marker Runx2 and for Sox2 was observed on the aortic side of the valves (Figure 4, bottom rows). Sox2 may have an emerging role in EC transitions, and was reminiscent of the *Mgp*^–/–^ aortas. Thus, the loss of MGP was associated with enhanced BMP activity and possible EC transitions.

## Perspectives

Altogether, there is increasing evidence of endothelial involvement in VC and CAVD. The ECs may participate in the calcific process through direct transition to mesenchymal and osteogenic cell lineages, secretion of pro-calcific growth factors, proteolytic activity that disrupts the IEL, induction of endothelial alkaline phosphatase, and inappropriate interactions with underlying cells (Figure 2; schematic figure). In addition, the ECs may be recruited into angiogenic networks that support mineralization, whereas circulating EPCs might contribute abnormal ECs during pro-calcific conditions.

The role of the endothelium in VC and CAVD may be conceptually similar, but transcriptionally distinct, each derived from separate vascular structures. Exploration of the heterogeneity in the two types of ECs could provide an advantage in designing exclusive treatments specific cardiovascular areas. The easy access to the endothelium from the circulation is an additional factor that would support therapeutic strategies aimed at the ECs.

Several areas of investigation are ripe for further exploration of the endothelial role in VC. For example, even though some connections between TGFβ and BMP signaling have been found in EndMTs, more information is needed. It is not clear how the two pathways relate in the early transitions or the emergence of osteoblast-like characteristics. The regulation of TGFβ and BMP receptors and ligands can be altered on several levels including formation of receptor-ligand complexes, transcriptional changes involving the SMADs that are common for both signaling systems, and the choice of using canonical versus non-canonical TGFβ/BMP signaling.

Furthermore, it would be important to know if ECs are required to undergo full or partial EndMT before acquiring enough of an osteogenic phenotype to influence VC, and if the transitions can be reversed at intermediate stages. Partial EndMTs might enhance plasticity and regeneration in the vasculature and accommodate changes without the ECs losing their basic lineage differentiation.

We also need a better understanding of the interactions between the vascular layers, and how the cells of the layers communicate in absence and presence of IEL or other barriers. We would need to know how the destruction of the IEL changes the interactions between ECs and medial cells and the susceptibility to EndMTs. We may have to change our viewpoint from considering individual cells to viewing all the vascular layers as a biological system, where the layers act as entities and the interactions rather than the cells are the targets. It might also be useful to know the transcriptional regulation of cell-specific proteases used to break down various barriers.

## Author Contributions

LZ methodology, investigation, and writing of manuscript. JY methodology and investigation. YY conceptualization, methodology, and investigation. KB conceptualization, writing of manuscript, supervision, and project administration. All authors contributed to the article and approved the submitted version.

## Conflict of Interest

The authors declare that the research was conducted in the absence of any commercial or financial relationships that could be construed as a potential conflict of interest.

## Abbreviations

ALK: Activin receptor-like kinase
ApoE: Apolipoprotein E
bFGF: Basic fibroblast growth factor (also known as FGF2)
BMP: Bone morphogenetic protein
BMPR2: BMP type 2 receptor
CAVD: Calcific aortic valve disease
CD: Cluster of differentiation (in CD31 and CD73)
Cdh5, Cadherin 5: VE-cadherin
CKD: Chronic kidney disease
DAPI: 4′,6-Diamidino-2-Phenylindole
EC(s): Endothelial cell(s)
EndMT: Endothelial-mesenchymal transition
EPC(s): Endothelial progenitor cell(s)
FOP: Fibrodysplasia ossificans progressiva
Gla: Gamma-carboxyglutamic acid
HDL: High-density lipoproteins
HUVEC(s): Human umbilical vein endothelial cell(s)
IEL: Internal elastic lamina
IL: Interleukin
LDL: Low-density lipoproteins
MGP: Matrix Gla protein
MMP: Matrix metalloproteinase
NT5E: Ecto-5′-nucleotidase
OCN: Osteocalcin
oxLDL: Oxidized low-density lipoproteins
ROS: Reactive oxygen species
Runx2: Runt-related transcription factor 2 (also known as core-binding factor subunit alpha-1 Cbfa1)
SMA: smooth muscle actin
SMAD: Small mothers against decaplentaplegic
SMC(s): Smooth muscle cell(s)
SMOC2: SPARC Related Modular Calcium Binding 2
Sox2: SRY (sex determining region Y)-box
TGF: Transforming growth factor
Tie2: TEK receptor tyrosine kinase
TNAP: Tissue-specific alkaline phosphatase
TNF: Tumor necrosis factor
VC: Vascular calcification
VEC(s): Valvular endothelial cell(s)
VIC(s): Valvular interstitial cell(s)
VEGF: Vascular endothelial growth factor
vWF: von Willebrand Factor.
